# The Role of miRNAs 340-5p, 92a-3p, and 381-3p in Patients with Endometriosis: A Plasma and Mesenchymal Stem-Like Cell Study

**DOI:** 10.1155/2021/5298006

**Published:** 2021-09-29

**Authors:** Afshin Bahramy, Narges Zafari, Pantea Izadi, Fatemeh Soleymani, Saeideh Kavousi, Mehrdad Noruzinia

**Affiliations:** ^1^Department of Medical Genetics, Faculty of Medical Sciences, Tarbiat Modares University, Tehran, Iran; ^2^Department of Medical Genetics, School of Medicine, Tehran University of Medical Sciences, Tehran, Iran

## Abstract

**Background:**

Endometriosis is the most prevalent gynecological disease with elusive etiology. The mysterious entity and the lack of noninvasive diagnostic methods affect women's lives negatively. This study is aimed at finding the relationship between miR-340-5p, 92a-3p, and miR-381-3p and the pathogenesis of endometriosis in endometrial mesenchymal stem-like cells (eMSCs) of endometriosis and assessing their potential as a noninvasive biomarker in plasma.

**Methods:**

Peripheral blood and eMSC specimens were collected from suspected women of endometriosis before laparoscopy. Total RNA was isolated from plasma and cultured eMSCs to synthesize complementary DNA. The expression of miR-340-5p, miR-92a-3p, and miR-381-3p was analyzed by RT-qPCR. To understand these miRNAs' role, we also did a bioinformatic analysis.

**Results:**

There was a downregulation of miR-340-5p, miR-92a-3p, and miR-381-3p in plasma, and the upregulation of miR-340-5p and the downregulation of miR-92a-3p and miR-381-3p in eMSCs of women with endometriosis. There was a positive concordance between the expression of miR-92a-3p and miR-381-3p in plasma and eMSCs. Our study also showed three genes, Solute Carrier Family 6 Member 8 (SLC6A8), Zinc Finger Protein 264 (ZNF264), and mouse double minute 2 (MDM2), as common targets of these miRNAs.

**Conclusions:**

This study has been one of the first attempts to examine the expression of miR-340-5p, miR-92a-3p, and miR-381-3p in both plasma and eMSCs and revealed their possible role in endometriosis based on in silico analysis. Biomarkers pave the way to develop a new therapeutic approach to the management or treatment of endometriosis patients. Our result as a first report shows that combined levels of miRNAs 340-5p and 381-3p may have the potential to be utilized as diagnostic biomarkers for endometriosis.

## 1. Introduction

Endometriosis is a chronic, inflammatory, and estrogen-dependent disease known as one of the most prevalent gynecological diseases that affects about 6-10% of women of reproductive age and close to 35-50% of infertile women [1, 2]. Endometriosis can be asymptomatic in 20-25% of cases, but it has some common symptoms such as chronic pelvic pain, dysmenorrhea, and dyspareunia [3]. Due to the lack of specific clinical manifestations, common symptoms with other gynecological disorders, and unknown etiology, endometriosis is not easy to diagnose; therefore, it negatively affects the quality of women's lives and their fertility status. Laparoscopy is currently used as the common method for definitive diagnosis of endometriosis followed by histopathological confirmation [4]. Besides the invasive entity of laparoscopy, it has some disadvantages, such as the need for general anesthesia and financially putting weight on the health system [5]. Moreover, among women who are diagnosed by laparoscopy, only 70-75% of them are histologically confirmed to have endometriosis [6]. Thus, the current diagnostic method has proven unreliable; therefore, considerable attention is drawn to find and validate (a) reliable biomarker(s).

MicroRNAs (miRNAs) are posttranscriptional regulators of gene expression with approximately 22 nucleotides that negatively mediate gene expression [7]. Besides their role in endometriosis's pathogenesis, miRNAs' potential as biomarkers has become an interesting topic and is extensively investigated due to their stable presence in body fluid, including plasma [8, 9]. Although investigations of endometriosis-specific miRNAs have been done in various body fluids, unfortunately, one or a panel of miRNA has not been identified yet to be utilized as a diagnostic biomarker. Consequently, because of the lack of (a) valuable noninvasive biomarker(s), the diagnosis of endometriosis might happen with approximately seven years delay from the onset [5], so the development of noninvasive biomarkers for the detection of endometriosis has received considerable attention among researchers.

The etiology of endometriosis remains elusive, and several theories about the origin of endometriosis have been proposed; among them, Sampson's theory is the most acceptable one [10]. Retrograde menstruation is a usual process and occurs in 70-90% of women; however, only about 10% of them experience endometriosis. So, this incident led to the belief that other factors, such as the immune system incompetency or alteration of the genetic and epigenetic processes, might play a role in the development of endometriosis [11]. Therefore, investigating mechanisms involved in endometriosis's pathogenesis is a critical issue because it would make it easier to diagnose and manage the disease. Observing a 7% increase in the incidence of endometriosis in first-degree relatives of the patients and a higher concordance rate in monozygotic than dizygotic twin pairs is another concrete evidence to consider endometriosis as a multifactorial disease [12, 13]. In addition, existing research recognizes that mesenchymal stem cells play a critical role in endometriosis's progress because of their self-renewal characterization. It enables them to overproliferate if they contain dysregulated stemness genes and miRNAs [14].

The studied miRNAs in this article were detected in a discovery phase study, and they showed significantly altered expression between endometriosis and nonendometriosis patients in our previous work (the article has not been published yet. The data were deposited in NCBI's Gene Expression Omnibus [15] and are accessible through GEO Series accession number GSE153813 (https://www.ncbi.nlm.nih.gov/geo/query/acc.cgi?acc=GSE153813/)). This study is aimed at investigating and determining the expression of miR-340-5p, miR-381-3p, and miR-92a-3p in the plasma and mesenchymal stem-like cells of endometriosis patients in an independent population to validate their expression status and examine their potential as noninvasive biomarkers and their possible role in endometriosis' pathogenesis, respectively.

## 2. Methods and Materials

### 2.1. Bioinformatic Analysis

The potential target genes of the studied miRNAs were predicted using miRNet (https://www.mirnet.ca/, a web-based tool that used several miRNA databases to get both predicted and experimentally validated target genes for miRNAs such as miRTarBase and TargetScan), miRTargetLink Human (https://www.ccb.uni-saarland.de/, which contain experimentally validated interactions of miRTarBase, and predicted targets were generated with miRanda), and Mienturnet (http://userver.bio.uniroma1.it/apps/mienturnet/, is a tool that was using computationally predicted or experimentally validated miRNA-target interactions from TargetScan). Then, the most commonly predicted target genes of microRNAs between the miRTargetLink, miRNet, and Mienturnet were screened by the Venn diagram (http://bioinformatics.psb.ugent.be/webtools/Venn/). The biological pathway and tissue protein expression analyses were carried out by EnrichR (https://maayanlab.cloud/Enrichr/), the web-based software with many gene set libraries, such as KEGG, Wikipathway, and Tissue Protein Expression from Human Proteome Map.

This project consists of two parts: (1) analysis of miRNA expression in plasma of endometriosis patients to identify their potential capability as a noninvasive biomarker; (2) assess their expression in mesenchymal stem cells of endometriosis patients as an influential pathological factor. All participation had written informed consent, and the Research Ethics Committee of TMU approved the study protocol.

### 2.2. Biomarker Detection Section

#### 2.2.1. Patients

Eighty volunteers of reproductive age (14-45 years old), who were referred to the infertility clinic of Imam Khomeini hospital affiliated with Tehran University of Medical Science (TUMS), were included in this study from 2018 through the year. All women were suspected of endometriosis because having one or a combination of symptoms included infertility, dysmenorrhea, and pelvic pain. Patients underwent laparoscopy to definite diagnose, and any possible ectopic tissue was sent for pathological confirmation. The stage of endometriosis was defined according to the American Society of Reproductive Medicine (ASRM) revised system (Revised American Society for Reproductive Medicine, 1997). The menstrual cycle phases of all patients were determined as the follicular phase (cycle days 6–15) and the luteal phase (cycle days 16–28) with regard to a 28-day cycle. The women with chronic inflammatory diseases, postmenopausal women, and women with a history of hormone medication consumption were excluded from our study.

#### 2.2.2. Sample Collection

The EDTA-containing tubes were used to collect whole blood samples (10 ml) before anesthesia and surgery. In less than 1 hour, the whole blood was transferred immediately to the laboratory and centrifuged at 1500 rpm and 15000 rpm subsequently for 15 min at 4°C. Then, the quality of plasma regarding RBC hemolysis was assessed by NanoDrop 2000 at 414 nm. Samples contained a high grade of RBC hemolysis were excluded from our study. Plasma with acceptable quality was aliquoted and stored at 80°C until further use.

### 2.3. Epigenetic Pathogenesis Section

#### 2.3.1. Sample Sources

Eutopic endometrial tissue samples were collected from women of reproductive age (20-45 years old) who underwent laparoscopy and hysterectomy in the Rasoul Akram Hospital of Iran Medical University (Tehran, Iran). Three endometriosis and three nonendometriosis patients were included in this study based on their surgery and histopathological examination results. All patients had not received any hormonal therapy for at least six months.

#### 2.3.2. Isolation and Culture of eMSCs

The eutopic endometrial tissue samples were harvested and washed in phosphate-buffered saline (PBS). Then, tissues were minced into 1-2 mm pieces in DMEM/F-12 medium (Invitrogen, UK) and 1% of penicillin-streptomycin antibiotics solution (Invitrogen, USA). The cell suspension was obtained from enzymatically (300 *μ*g/ml of collagenase type *ΙΙΙ*, Sigma, Germany) and mechanically digested tissue (incubation at 37°C for 90 minutes), then was centrifuged for 5 minutes at 3000 rpm, and was filtered through 150, 100, and 40 mm mesh to achieve pure eMSCs without any components. eMSCs were cultured in DMEM/F-12 medium, containing 1% penicillin-streptomycin solution and 10% fetal bovine serum (FBS, Gibco, USA). In the next step, cultures were incubated at 37°C in 95% air and 5% CO_2_ concentration. The cultures underwent three passages to be prepared for the next step.

#### 2.3.3. Flow Cytometry Assessment of eMSCs

Mesenchymal cells were digested enzymatically by trypsin and centrifuged to obtain the cell pellet, which was resuspended again in 5% FBS and incubated in a dark place for 30 minutes at 4°C in monoclonal antibodies. Negative control antibodies (human CD45, BD Biosciences, and CD34, IMMUNOSTEP, Spain) and specific antibodies (anti-human CD90 (BD Biosciences, USA), CD105 (IMMUNOSTEP, Spain), CD73 (BD Biosciences, USA), and CD146 (BD Biosciences, USA)) were used to cells' assessment by a FACS Calibur apparatus (Becton Dickinson, USA).

#### 2.3.4. The Potential of eMSCs' Differentiation

eMSCs were cultured in a medium consist of osteogenic and adipogenic differentiation separately for four weeks. To culture the control cells, DMEM/F12 medium (consist of 1% FBS and 1% penicillin-streptomycin antibiotic solution) was used in the same incubation time. Then, 4% alizarin red stain (pH = 4.1) (Sigma, Germany) and 1% Oil Red O stain (Sigma, Germany) were used to stain the culture and assessed the osteogenic and adipogenic differentiation.

### 2.4. RNA Isolation

According to the manufacturer's protocol, 400 *μ*l plasma and cells were used for RNA extraction using an RNX-PLUS reagent (CinnaClone, Iran) in a final volume of 20 *μ*l. Eventually, the quality and quantity of RNA were determined using a NanoDrop 2000 (the ratio of absorbance at 260/280 and 260/230 nm ≥ 1.8).

### 2.5. RT-qPCR

Isolated RNA (100 ng/*μ*l) was used to synthesize complementary DNA (cDNA) of miR-340-5p, miR-381-3p, and miR-92a-3p with a Reverse Transcription Kit (GeneAll, Korea). In reverse transcription (RT), the specific stem-loop primers of each miRNA were used to increase the detection's sensitivity and specificity. In this study, we used the universal stem-loop sequence proposed by Chen et al. for the first time [16]: 5′-GTCGTATCCAGTGCAGGGTCCGAGGTATTCGCACTGGATACGAC-3′. The stem-loop sequence, which was used specifically for each miRNA, is provided as follows:

mir-16-5p 5′-GTCGTATCCAGTGCAGGGTCCGAGGTATTCGCACTGGATACGACCGCCAA-3′; miR-92a-3p 5′-GTCGTATCCAGTGCAGGGTCCGAGGTATTCGCACTGGATACGACACAGGC-3′; miR-340-5p 5′-GTCGTATCCAGTGCAGGGTCCGAGGTATTCGCACTGGATACGACAATCAG-3′; miR-381-3p 5′-GTCGTATCCAGTGCAGGGTCCGAGGTATTCGCACTGGATACGACACAGAG-3′.

RT-qPCR was conducted by SYBR Green RealQ Plus 2x Master Mix Green (Ampliqon, Denmark) using the Applied Biosystems StepOne Real-Time PCR System (Life Technologies, USA) with the specific forward primers to miR-340-5p, miR-381-3p, and miR-92a-3p, and the universal reverse primer complementary to the stem-loop primer. miR-16 was selected as an internal control to normalize the expression level of miRNAs. The previous studies represented that miR-16 expression is stable and less variable in plasma [17]. RT-qPCR reaction mixture included 2 *μ*l of cDNA, 0.5 *μ*l of forward primer (10 pmol), 0.5 *μ*l of reverse primer (10 pmol), 5 *μ*l of SYBR Green PCR Master Mix, and 2 *μ*l of nuclease-free water. Reactions were conducted at 95°C for 30 s at incubation time, followed by 40 cycles of amplification at 95°C for 10 s, annealing at 60°C for 15 s, and extension at 72°C for 30 s. At the end of the process, melting curve analyses were carried to confirm our RT-qPCR amplification specificity.

All reactions were performed in duplicate, and we used a 2^-*ΔΔ*Cq^ formula to analyze the raw data of RT-qPCR, and all expression was normalized to internal control miRNA. The universal reverse primer and the specific forward sequence are provided in the following, respectively.

Universal reverse primer: 5′-AGGGTCCGAGGTATTCGC-3′, specific forward sequence of has-miR-16-5p 5′-GAGGGTAGCAGCACGTAAAT-3′; hsa-miR-92a-3p 5′-CGTATTGCACTTGTCCCGC-3′; has-miR-340-5p 5′-GGCGGTTATAAAGCAATGAGT-3′; hsa-miR-381-3p 5′-CAGGTATACAAGGGCAAGCT-3′.

### 2.6. Statistical Analysis

Data were expressed as mean ± standard deviation (SD), or proportions were appropriate. Student's *t*-test was used to determine the difference between clinical characteristics of endometriosis and nonendometriosis patients. The Kolmogorov-Smirnov test was used to assess the normal distribution of variables related to plasma. The levels of plasma miRNA expression between groups were compared using the nonparametric Mann-Whitney *U* test for nonnormally distributed data (*P* value < 0.05) and the parametric *t*-test for normally distributed data (*P* value > 0.05) to compare the studied variables. IBM SPSS Statistics 22 (SPSS Inc., Chicago, IL, USA) was used for statistical analyses. A *P* value < 0.05 represents statistically significant differences between groups. ROC curve (AUC) was performed to determine the diagnostic potential of miRNA expression for each miRNA separately and in combinations. The best statistical cut-off values of miRNA expression levels and then sensitivity and specificity for selected cut-off points were assessed according to the ROC curve. In eMSCs, the Shapiro-Wilk test was used to analyze the normal distribution of data. We performed one-way ANOVA to assess significance level miRNA expression, and *P* value < 0.05 was considered a statistically significant difference. IBM SPSS Statistics 22 (SPSS Inc., Chicago, IL, USA) was used for all measurements. GraphPad Prism 8 (GraphPad Software Inc., San Diego, CA, USA) was used to draw bar charts in this study.

## 3. Results

### 3.1. Bioinformatic Analysis

**Figure 1:**
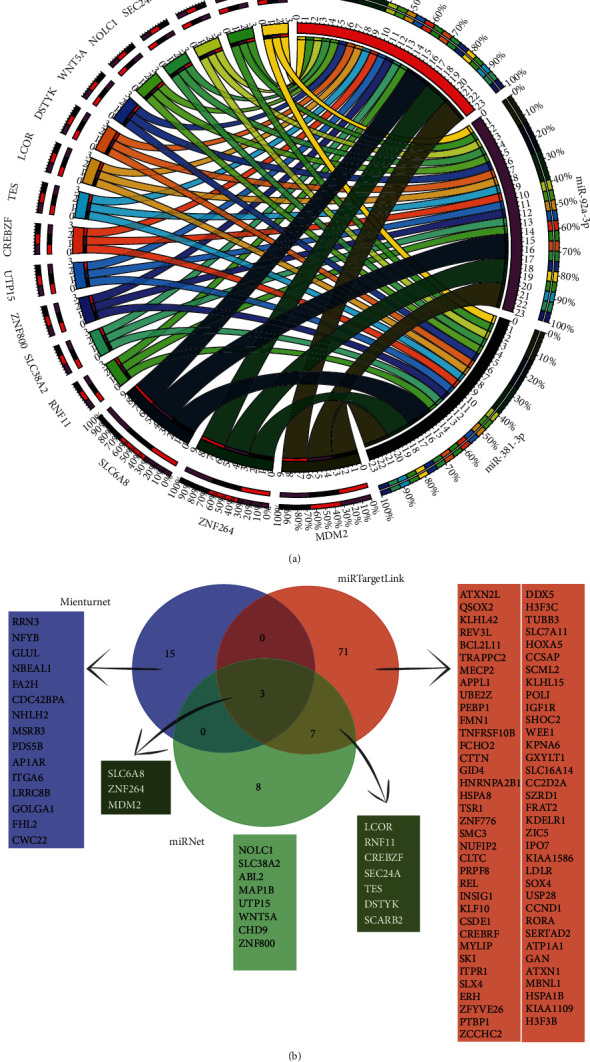
Target genes overlap between three miRNAs. Circos plot shows miRNA-mRNA interactions; 17 common genes are targeted by three miRNAs—miR-340-5p, miR-92a-3p, and miR-381-3p. Ribbons start from an mRNA (leftmost) and end in a miRNA (rightmost); the color of the ribbons indicates the originating mRNAs. Ribbon thickness indicates which mRNAs interact the most with all three miRNAs; the outermost ring displays miRNA color (leftmost) (a). A Venn diagram representing the number of miRNAs' target genes in common between miRNet, miRTargetLink, and Mienturnet (b).

The three candidate miRNAs in this study were subject to further bioinformatic analyses to identify the most commonly predicted target genes. Web-based tools (miRNet, miRTargetLink Human, and Mienturnet) were used to provide targets of the combined miRNAs to predict the putative target genes of the candidate miRNAs. Using miRTargetLink, 81 target genes were found, miRNet revealed 18 target genes, and by using Mienturnet, 18 target genes were found for a combination of miRNAs (Figure 1). Interaction networks to predict miRNAs' target genes in common were created and shown by the Circos plot (Figure 1(a)). Besides, Venn diagram analyses revealed that SLC6A8, ZNF264, and MDM2 were common as potential miRNAs' targets in the miRtargetLink, miRNet, and Mienturnet (Figure 1(b)).

**Figure 2:**
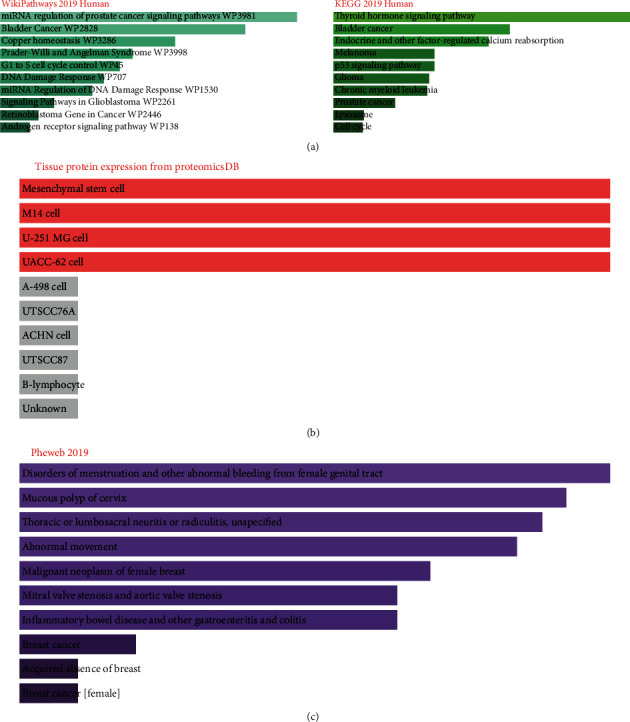
Gene set enrichment analysis performed by EnrichR of target genes of miR-340-5p, miR-381-3p, and miR-92a-3p in endometriosis. Top 10 enriched pathways by WikiPathways 2019 Human and KEGG 2019 Human correlated with miRNAs' target genes. The *x*-axis represents the number of genes, and the *y*-axis represents enriched pathway (a). Gene set enrichment analysis by EnrichR showed the top 10 enriched correlated miRNA's target genes in the library of Tissue Protein Expression from Human ProteomicsDB (b). Gene set enrichment analysis by EnrichR showed the top 10 enriched correlated miRNA's target genes in the PheWeb 2019 (c).

Next, we used the EnrichR platforms to identify the cellular and molecular pathways potentially regulated by the miRNAs' target genes in this study. KEGG and WikiPathways analysis assigned several well-documented pathways related to endometriosis according to previous studies [18–20]: P53 signaling pathway, androgen receptor signaling pathway, copper homeostasis, DNA damage response (DDR), and cell cycle (Figure 2(a)). Based on the gene set library of Tissue Protein Expression from ProteomicsDB, common genes between three miRNAs in this study enriched in a mesenchymal stem cell are shown to have a critical role in the pathogenesis of endometriosis (Figure 2(b)) [21]. Moreover, gene set enrichment analysis by EnrichR based on PheWeb showed that the positively correlated target genes of these three miRNAs were mainly associated with menstruation disorders (Figure 2(c)), which in women with endometriosis disrupted and had a higher rate of abnormal menstrual scores than those without the disease [22]. This could explain the retrograde theory, the most popular theory of endometriosis's pathogenesis [23].

### 3.2. Quality Assessment of the Plasma

In total, eighty plasma samples were collected in the first step, which was analyzed by NanoDrop 2000 to determine whether there was any RBC hemolysis or not. Plasma samples with OD ≥ 0.3 were excluded from our study because of significant RBC hemolysis, and ones with OD < 0.3 were included. Therefore, based on this reason, as mentioned earlier, we did not use twenty of the collected plasma sample and put them aside.

### 3.3. Clinical Characteristic

The clinical information of participants in the biomarker detection section is summarized in Table 1. The definite diagnoses of each patient were surgically and histologically confirmed. The mean age (mean ± SD) of endometriosis and nonendometriosis patients was 33.6 ± 1.15 and 31.4 ± 1.23 years, respectively. Thirty women in the endometriosis group suffered from stage III (*n* = 18) and IV (*n* = 12). Thirty women in the nonendometriosis group had a benign gynecological disease such as myoma, fibroma, polyps, and ovarian cyst, and four of them were healthy women. Moreover, all participants' menstrual cycle was determined, and there was no statistically significant difference in its distribution among the case and control groups.

Endometrial tissues were collected from three women with endometriosis and three women without endometriosis. Their age was between 20 and 45 years. There was no sign of systemic or gynecological disease among healthy women, and endometriosis patients suffered from the advanced disease stage (III and IV). All participants had a regular menstrual cycle.

### 3.4. Expression of miR-340-5p, 92a-3p, and 381-3p in the Plasma of Endometriosis Patients Incomparable with Nonendometriosis Patients

**Figure 3:**
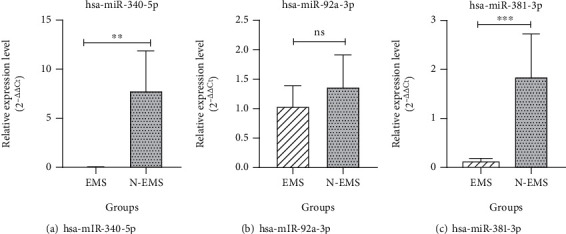
miRNAs' expression in the plasma of endometriosis patients (EMS) and nonendometriosis patients (N-EMS) was assessed by RT-qPCR. The *P* value shows statistical significance of results between the case and control groups used for variables with nonnormal distribution: miR-340-5p (a) and normal distribution miR-92a-3p (b) and miR-381-3p (c); *n* = 60 (30 endometriosis patients; 30 nonendometriosis patients) (US: insignificant, *P* value: ^∗^*P* value < 0.05, ^∗∗^*P* value < 0.01, and ^∗∗∗^*P* value < 0.0001).

The relative expressions of miR-340-5p, 92a-3p, and 381-3p in the plasma of endometriosis and nonendometriosis patients were assessed using RT-qPCR. Each miRNA expression is shown in Figure 3. Of which, the miR-381-3p and 340-5p expression were significantly lower in the endometriosis vs. nonendometriosis patients (*P* value ≤ 0.01 and 0.009, respectively), while lower expression of 92a-3p was not statistically significant (*P* value ≤ 0.205).

### 3.5. Assessment of Circulating miR-340-5p, 92a-3p, and 381-3p Expression according to the Menstrual Cycle and Endometriosis' Stages of Patients

To evaluate the potential menstrual cycle's effect on these miRNAs' expression patterns, we assessed the level of miRNA expression in the follicular phase compared to the luteal phase of patients without considering their group. In addition, miRNAs' expression level was analyzed in the follicular phase and the luteal phase of endometriosis patients vs. nonendometriosis. Eventually, there was no significant association between miR-340-5p, 92a-3p, and 381-3p expression and the menstrual cycle (*P* value > 0.05). Furthermore, we analyzed the expression of each miRNA based on the stage of endometriosis to demonstrate if there was a possible change, and we found no significant variation in miRNAs' expression among these stages (*P* value > 0.05).

### 3.6. Estimation of the Diagnosis Value of the miRNAs Separately and as a Panel

**Figure 4:**
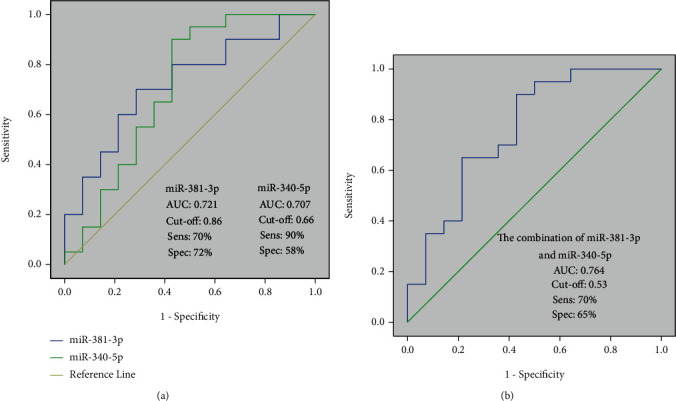
The results of ROC curve analysis for the diagnostic value of miRNAs 340-5p and 381-3p (a) and their combination (b), the area under the curve (AUC) 0.707, 0.721, and 0.764, respectively.

We performed the ROC curve analysis of differentially expressed miRNAs in plasma to assess their utility as a diagnostic biomarker. The AUC of miR-340-5p and miR-381-3p were 0.707 (95% CI: sensitivity = 90% and specificity = 58%) and 0.721 (95% CI: sensitivity = 70% and specificity = 72%). Furthermore, the AUC of their combination as a diagnostic panel to differentiate two groups was 0.764 (95% CI: sensitivity = 70% and specificity = 65%) (Figure 4).

### 3.7. Isolation and Characterization of eMSCs

After three passages, flow cytometry analysis of eMSCs was positive for mesenchymal stem cell (CD73 (98.5%), CD90 (99.1%), and CD105 (96.3%)), endometrial stem cell (CD146 (84.8%)), and negative for hematopoietic (CD34 (0.474%) and CD45 (1.99%)) markers. Finally, the potential of endometriotic MSCs to differentiate was confirmed by the staining of calcium deposits and lipid vacuoles using alizarin and oil red receptively. The details clearly explained the result of flow cytometry in the previously published study [14].

### 3.8. Expression of miR-340-5p, 92a-3p, and 381-3p in eMSCs

**Figure 5:**
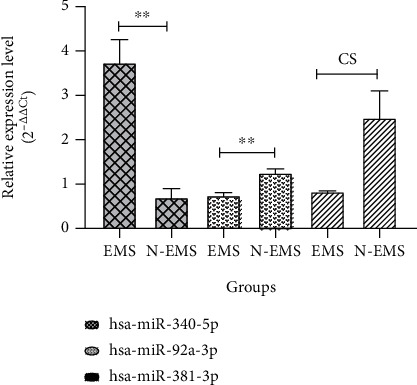
miRNAs' expression in the eMSCs of endometriosis patients (EMS) and nonendometriosis patients (N-EMS) was assessed by RT-qPCR. The *P* value shows statistical significance of results between the case and control groups used for variable normal distribution; *n* = 6 (3 endometriosis patients; 3 nonendometriosis patients) (CS: close to significant, *P* value: ^∗^*P* value < 0.05, ^∗∗^*P* value < 0.01, and ^∗∗∗^*P* value < 0.0001).

The relative expression of miR-340-5p, 92a-3p, and 381-3p in eMSCs of endometriosis compared to nonendometriosis patients was assessed by RT-qPCR. The expression level of miR-92a-3p (*P* value ≤ 0.008) and miR-381-3p (*P* value ≤ 0.051) was downregulated, and miR-340-5p (*P* value ≤ 0.005) expression was upregulated in endometriosis patients (Figure 5).

## 4. Discussion

### 4.1. Pathogenesis Involvement Section Based on Bioinformatic Analysis

Bioinformatic analysis revealed three genes, mouse double minute 2 (MDM2), Solute Carrier Family 6 Member 8 (SLC6A8), and Zinc Finger Protein 264 (ZNF264), as the most commonly predicted target genes of miR-340-5p, miR-381-3p, and miR-92a-3p.

The role of MDM2 as an important gene that regulates apoptosis was shown in the pathogenesis and development of endometriosis [24]. The overexpression of MDM2 was previously reported as a contributor to endometriosis progression by promoting proliferation, migration, and invasion and inhibiting apoptosis [25]. Another study by Liu et al. revealed the axis linking MDM2 to endometriosis pathogenesis via miR-610 in the ectopic endometrial stromal cells, which introduced MDM2 as a potential therapeutic target for endometriosis [26]. The SLC6A8 gene is a creatine transporter that shows overexpression during the secretory phase in stromal cells and endometrial glands [27]. The SLC6A8 gene has been identified as a circulating tumor cell marker for gynecological malignancies such as endometrial cancer [28]. The ZNF264 gene has not been investigated in many studies, so its role in endometriosis and other disorder is still unclear; however, it was mentioned that ZNF264 has an oncogenic role in signaling transduction invasion/metastasis [29], which are important pathway involved in endometriosis [30].

Based on gene set enrichment analysis by EnrichR, miRNAs' target genes were enriched in the P53 signaling pathway and cell cycle, which play an important role in apoptosis [20]. Additionally, our analysis suggested that the androgen receptor signaling pathway is a potential target gene of our selected miRNAs. This observation suggests that the AR receptor may play a role in the cause of the endometriosis' pathogenesis, which was previously reported in endometrial cancer and other gynecological disorders [20]. DNA damage response (DDR) is another identified pathway; it has been explained that genes involved in DDR dysregulated in women with endometriosis compared to those without the disease [18]. Copper homeostasis was also among the pathways that EnrichR analysis predicted as a pathway involving the target genes of selected miRNAs. This could be of particular interest since cooper (CU) was associated with endometriosis etiopathogenesis [19]. Finally, the pathway analysis found that the cell cycle, alongside DDR, might be among the pathways controlled by miRNAs' target genes in endometriosis [20].

So, these genes may have a role in endometriosis's pathogenesis, but further investigations need to reveal their role precisely.

### 4.2. Biomarker Detection Part

As a disease with nonspecific symptoms that might also be asymptomatic, endometriosis remains difficult to diagnose [31]. Relatively small endometriosis lesions are mostly found in the peritoneal cavity, so laparoscopy with histological confirmation is still the common diagnosis method [5]. Due to the invasive entity of laparoscopy and lack of biomarkers, the detection of endometriosis postpones almost seven years [5]. Since epigenetics has been a critical factor in the pathogenesis of endometriosis, the researcher's attention was drawn to miRNAs as a potential biomarker [32]. miRNAs are easy to detect without serious damage because their expression is stable in plasma, and they show minor changes when it comes to variables such as age, gender, body mass index (BMI), and smoking status [33].

Here, we performed RT-qPCR to investigate the expression levels of miR-340-5p, 92a-3p, and 381-3p in patients with endometriosis for providing new insights towards the circulating biomarkers for endometriosis diagnosis and management in clinical practice. The present study showed a significantly lower expression of miR-340-5p and miR-381-3p, but there was no significant altered expression related to miR-92a-3p in the plasma of endometriosis patients, although its expression was downregulated. Remarkably, their altered expression levels were independent of the menstrual cycle and endometriosis stage of the participants. Furthermore, to evaluate the utility of the identified miRNAs as diagnostic biomarkers for endometriosis, ROC curves were used. The diagnostic accuracy of miR-340-5p indicated a sensitivity and specificity of 90% and 58%, respectively, at a cut-off value of 0.66; furthermore, the 70% sensitivity and 72% specificity at a cut-off value of 0.86 was assessed for miR-381-3p. Using the logistic regression model, the diagnostic value of the combination of miR-340-5p and miR-381-3p was assessed and yielded in AUC of 0.764. Alongside our results, previous studies also reported the altered expression of these miRNAs in various body fluids. Downregulation of miR-340-5p (plasma), miR-381-3p (serum), and miR-92a-3p (plasma) has already been reported in the endometriosis patients, but only the diagnostic value of miR-340-5p was determined with 88% sensitivity and 53% specificity at a cut-off value of 0.24 [17, 34, 35].

Although CA-125 and ultrasound have been recognized as noninvasive methods to detect endometrial lesions, their flaws are not deniable. CA-125 is not specific to endometriosis, and it also alters in other relevant gynecological conditions such as leiomyoma and chronic inflammation of the pelvic [36]. CA-125 showed a specificity of 96% and sensitivity of 57%, which are not adequate to use as a definite noninvasive biomarker [37]. Transvaginal ultrasound can reliably diagnose endometrial lesions with more than 90% sensitivity and specificity. Magnetic resonance imaging (MRI) has 94% sensitivity and 79% specificity, which is relatively low [38]. On the other hand, it has been proved that both CA-125 and transvaginal ultrasound are only useful for detecting endometriosis at advanced stage (III and IV) [38, 39].

Notwithstanding the relatively limited specificity of miR-340-5p, interestingly, our study showed 90% sensitivity for this miRNA, a high true positive value, which means that 90% of actual endometriosis patients are correctly identified as positive. Moreover, numerous studies showed miRNAs' potential as suitable biomarkers [40]. A study assessed a panel of miR-362-5p, 628-3p, and 1915 as a diagnostic biomarker and showed it is capable of distinguishing endometriosis patients from control groups with AUC 0.88, sensitivity 90%, and specificity 73% [41]. It also has been shown a combination of miR-199b-3p, miR-224-5p, and Let-7d-3p with 96% sensitivity and 100% specificity is another potential panel for noninvasive diagnose of endometriosis, which has higher diagnostic accuracy than CA-125, without any dependence on the menstrual phase or a particular stage of the disease [42]. Although there are studies that also showed the relatively unsuitable diagnostic value of miRNAs [34, 41], they could not definitely rule out miRNAs' competence as a potential noninvasive biomarker. So, miRNAs remain a promising target of biomarker detection studies in endometriosis and other benign and malignant diseases. However, it certainly takes time to validate a miRNA or a panel of them, to distinguish patients from healthy people in all populations with high sensitivity and specificity.

### 4.3. Pathogenesis Involvement Section Based on Previous Studies

Endometriosis is a gynecological disease identified by the growth of endometrial-like tissue outside the uterus [1]. Despite the high prevalence of endometriosis and its effect on women's quality of life, endometriosis's exact etiology is still unknown. As a most accepted mechanism, Sampson's theory cannot adequately explain what exactly puts women at a high risk of endometriosis incidence [11]. So, it is speculated that retrograde menstruation probably is not the only participating factor and biological behaviors of ectopic tissue such as migration and invasion have a key role in endometriosis's pathogenesis [43]. Increased endometrial, epithelial, and stromal cells suggest that the endometrium in this disease might have an enhanced ability to proliferate, implant, and survive [44]. Epithelial-to-mesenchymal transition (EMT) is also considered a possible mechanism of endometriosis's pathogenesis [45]. Highly regenerative endometrial tissue consists of two kinds of cells, epithelial cells and mesenchymal cells (stromal cells), and functional endometrium consists of the functionalis layer and the basalis layer. During each menstrual cycle, the whole functional layer and, to a lesser extent, the basalis layer shed and renew. Progenitor stem cells and probably mesenchymal stem cells (MSCs) which reside in the basalis layer are responsible for this ability [46]. Indeed, it is the growth, development, differentiation, and reprogramming of overlying epithelium which are regulated by mesenchymal cells (stromal cells) [47]. During EMT, epithelial cells lose their features and turn into mesenchymal cells, which result in the loss of the mesothelial barrier as a protective barrier, cell-to-cell contacts, polarity and acquirement of the high mobility, invasiveness, and resistance to apoptosis [48]. As long as mesenchymal cells are increased, during the retrograde menstruation process, aberrant levels of MSCs, which are undifferentiated, can be shed through the fallopian tube and migrate out of the uterus to the ectopic sites and establish endometriotic lesions. One of the main reasons for EMT's occurrence and impaired functions of MSCs is miRNAs [14, 49].

We isolated eMSCs from eutopic endometrial tissues of endometriosis patients and assessed the expression of CD146 through flow cytometry analysis (84.8%), known as the human endometrial MSc marker. Alongside our results, previous studies have reported an increased level of CD146 marker in endometriotic mesenchymal stem cells (eutopic MSCs of endometriosis patients) [50]. CD146 is a perivascular cell marker, and CD146+ cells were also located perivascularly in both functionalis and basalis layers of the human endometrium. Therefore, it has been shown that this marker involves in angiogenesis and proliferation that are important pathways related to endometriosis's pathogenesis. [51].

We performed RT-qPCR to analyze the expression level of miR-340-5p, 92a-3p, and 381-3p in eMSCs of endometriosis patients. In our study, the expression level of miR-92a-3p and miR-381-3p was lower, while the expression level of miR-340-5p was higher in eMSCs of endometriosis patients.

Other studies indicated the downexpression of miR-340-5p in different cancers, such as prostate cancer [52]. It has been reported that the miR-340 is an intronic miRNA located in the RNF130 gene; because of the CpG island in the promoter of miR-340, it could be methylated and epigenetically silenced. This methylation was inversely associated with the expression of miR-340-5p [53]. Indeed, it has been reported that miR-340 is capable of reducing methylation by decreasing the expression of DNA methyltransferase 1 (DNMT1) [54]. DNMT1 is also shown to be downregulated in endometriosis's ectopic tissue compared to healthy endometrium [55]. So, it is supposed that miR-340 by targeting DNMT1 reduces the DNMT1 expression, and it would result in the hypomethylation of RNF130/miR-340 promoter and upregulation of miR-340 [54].

**Figure 6:**
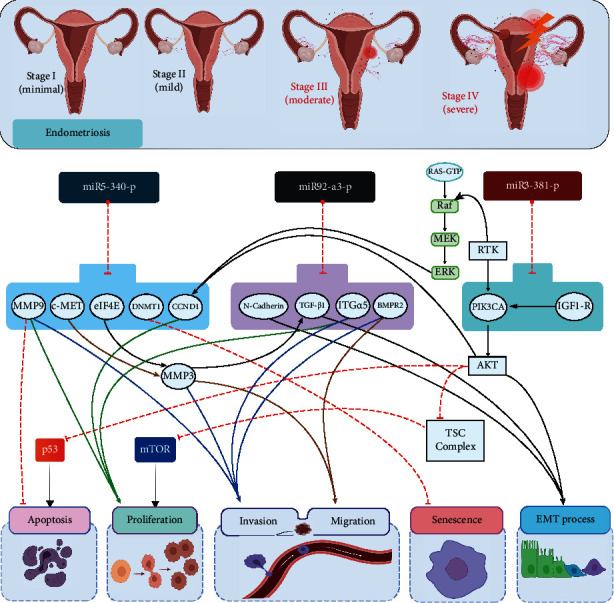
The endometriosis stages and the possible role of miR-340-5p, miR-92a-3p, and miR-381-3p in endometriosis, their target genes, and related pathways. MMP9: matrix metalloproteinase-9; CCND1: cyclin D1; DNMT1: DNA methyltransferase 1; PIK3CA: Phosphatidylinositol-4, 5-Bisphosphate 3-Kinase Catalytic Subunit Alpha; IGF-1R: insulin-like growth factor receptor; c-MET: MET protooncogene, receptor tyrosine kinase; eIF4E: eukaryotic translation initiation factor 4E; TGFB1: transforming growth factor beta 1; ITGA5: integrin subunit alpha 5; BMPR2: bone morphogenetic protein receptor type 2. The well-known process and pathways in endometriosis are apoptosis, proliferation, invasion, migration, senescence, and EMT process.

One of the direct targets of miR-340 is c-Met (tyrosine-protein kinase Met), a cell surface receptor tyrosine kinase known as a protooncogene [56]; c-Met is responsible for the mediation of cell migration and invasion through gelatinases (MMP2 and MMP9) [57]. MMP3 and MMP9 expression is also mediated by miR-340-5p, which influences the proliferation and migration of endometrial cancer cells [58]. It also has been reported that c-Met is a receptor of HGF (Hepatocyte Growth Factor) [56]. HGF is produced by endometrial stromal cells and has a role in proliferation, migration, and formation of endometrial cells [59]. miR-340-5p regulates the expression of p-eIF4E endometrial carcinoma cell, and p-eIF4E suppresses the TGFB1-induced EMT through MMP3 regulation [58, 60]. It has been suggested that miR-340 might play a potential role as a tumor suppressor in the regulation of stem-like cell activation when they reveal cancerous properties [54]. Altogether, the higher expression of miR-340-5p is correlated with lower cell proliferation and invasion (Figure 6).

We assume that the observation of overexpressed miR-340-5p in endometriosis patients, which is consistent with the role of it as a potential tumor suppressor, might play a protective role against the disease's progression or avoid the development of advanced stages of endometriosis and even the development of cancer, but it needs more investigation to prove.

Alongside our study, the lower expression of miR-92 has been shown previously in endometrial mesenchymal/stromal cells in endometriosis [61]. However, its higher expression was also found in various cancers such as colorectal adenoma [62]. Nevertheless, it has been shown that miRNAs can serve both an oncogenic or tumor-suppressing function based on their target genes [63]. miR-92a is a member of the miR-17-92 cluster that is located on chromosome 13q32-33 [64]. miR-17-92 cluster is known as oncomir-1 and responsible for increasing cell proliferation and decreasing apoptosis in lung cancer and lymphoma [64, 65]. Although there is much evidence to prove that miR-92 is an oncogenic miRNA [63], the existence of contradictory evidence such as loss of heterozygosity (LOH) at encoding locus of the miR-17-92 cluster that occurs in 16.5% of ovarian cancers, 21.9% of breast cancers, and 20% of melanomas revealed that this locus could act as a tumor suppressor [66]. Previous studies showed that the miR-17-92 cluster could undermine the self-renewal capacity of cancer stem cells and the occurrence of EMT through regulation of various genes such as NODAL/ACTIVIN/TGFB1 and N-cadherin, respectively [67, 68]. Lower expression of miR-92 also increases invasion, migration of breast cancer epithelial cells, and more aggressive tumor phenotype through upregulation of TGFBR2 and BMPR2 [63, 69]. It also has been shown the upregulation of miR-92 in breast cancer improves the survival rate and a better clinical outcome of patients comparable to those who had a lower expression [63, 69]. miR-92a is a negative regulator of angiogenesis by suppressing integrin *α*5 (ITGA5); therefore, it could impede the formation of the vascular network [70], and also, the overexpression of ITGA5 in ovarian cancer could result in tumor cell adhesion, metastasis, and proliferation [71].

In the present study, we also observed the downregulation of miR-92a in eMSCs of endometriosis, and all of the discussed mechanisms are relevant to its pathogenesis; however, only a few studies provided the molecular mechanism underlying the exact function of miR-92a as a tumor suppressor. Still, its mechanism in endometriosis has been elusive and needs further functional studies to determine.

### 4.4. Strengths and Limitations of the Current Study

One of the present study's outstanding strengths is that this study provides the first comprehensive assessment of the expression of miR-340-5p, miR-92a-3p, and miR-381-3p in both eMSCs and plasma of endometriosis patients, as well as performing bioinformatic analysis to revealed possible role of these miRNAs in endometriosis. Moreover, we used plasma instead of serum since the concentration of miRNAs is higher in plasma [72]. Furthermore, miR-16 was used as an internal control to normalize target miRNAs' expression levels since it is more stable than most frequently used housekeeping genes, including U6 small nuclear RNA or 5S ribosomal RNA [73]. Patients' selection in the biomarker detection section is also an advantage of our study because we choose women who suffered from the same symptoms as endometriosis patients. We aimed to find a biomarker to prevent unnecessary surgery in women who do not need laparoscopy as a treatment option. On the other hand, because of the high frequency of endometriosis, if we had chosen our control group from a healthy population, it would have been possible that undiagnosed or asymptomatic endometriosis patients existed among them.

Our study also had some limitations which deserve to be mentioned. We are aware that for the detection of diagnostic biomarkers, a larger prospective study is needed. In this context, further large-scale clinical studies with longitudinal data in different populations are needed to expand our knowledge about these miRNAs' generalizability for endometriosis diagnosis. In the pathogenesis involvement of miRNA section, the control group was chosen among healthy women who were a candidate for laparoscopy to uterine resection, not their health status, and we also did not compare the expression of these miRNAs among utopic and ectopic tissue of endometriosis patients, which could be a source of variation when it comes to miRNAs' expression.

## 5. Conclusions

Our results demonstrated altered expression levels of miR-340-5p, 92a-3p, and 381-3p in plasma and eMSCs of endometriosis patients. There was a concordance between the altered expression of 92a-3p and 381-3p in both target tissue. The different expression pattern of miR-340-5p in plasma and eMSCs of endometriosis suggests that the relationship between miRNA signature in body fluids and relevant tissues has regular patterns. Still, their origin and function in both body fluids and tissues must be determined. Although selected miRNAs in this study did not demonstrate a reasonable diagnostic accuracy for distinguishing endometriosis patients from nonendometriosis women, their altered expression in mesenchymal stem cells might happen due to their involvement in the pathogenesis of endometriosis or even exertion as protective factors against disease's progression or even cancer's development. They also jointly target three genes (SLC6A8, ZNF264, and MDM2) with a possible role in endometriosis pathogenesis. These findings provide the following insight for future research using functional studies, which could shed more light on these miRNAs' role as tumor suppressors and their roles in endometriotic MSCs.

## Figures and Tables

**Table 1:** Clinical characteristics of endometriosis and nonendometriosis patients contributed in biomarker detection in plasma.

	Endometriosis women (*n* = 30)	Nonendometriosis women (*n* = 30)	*P* value
*Age (years,*mean ± SD)	33.6 ± 1.15	31.4 ± 1.23	0.75
*Cycle phase,n(%)*			
Follicular	20 (66.6)	18 (60)	*P* > 0.05
Luteal	10 (33.3)	12 (40)	*P* > 0.05
*ARSM stage,n(%)*			
III	18 (60)	NA^∗^	NA
IV	12 (40)	NA	NA
*Other diagnoses in nonendometriosis women,n(%)*			
Healthy	NA	5 (16.6)	NA
Ovarian cysts	NA	9 (30)	NA
Myoma	NA	3 (10)	NA
Fibroma	NA	2 (6.6)	NA
Polyps	NA	3 (10)	NA
Other gynecological complication	NA	8 (26.6)	NA

^∗^NA = not applicable.

## Data Availability

Data generated and analyzed during this study are included in this published article and available from the corresponding author.
